# Method for manufacture and cryopreservation of cartilage microtissues

**DOI:** 10.1177/20417314231176901

**Published:** 2023-07-26

**Authors:** Md. Shafiullah Shajib, Kathryn Futrega, Rose Ann G Franco, Eamonn McKenna, Bianca Guillesser, Travis J Klein, Ross W Crawford, Michael R Doran

**Affiliations:** 1 School of Biomedical Science, Faculty of Health, Queensland University of Technology, Brisbane, QLD, Australia; 2 Centre for Biomedical Technologies, School of Mechanical, Medical, and Process Engineering, Faculty of Engineering, Queensland University of Technology, Brisbane, QLD, Australia; 3 Translational Research Institute, Brisbane, QLD, Australia; 4 National Institute of Dental and Craniofacial Research, National Institutes of Health, Department of Health and Human Services, Bethesda, MD, USA; 5 Mater Research Institute – University of Queensland, Brisbane, QLD, Australia

**Keywords:** Cartilage, cryopreservation, microtissue, organoid, tissue engineering, microwell, chondrocyte, mesenchymal stem cell

## Abstract

The financial viability of a cell and tissue-engineered therapy may depend on the compatibility of the therapy with mass production and cryopreservation. Herein, we developed a method for the mass production and cryopreservation of 3D cartilage microtissues. Cartilage microtissues were assembled from either 5000 human bone marrow-derived stromal cells (BMSC) or 5000 human articular chondrocytes (ACh) each using a customized microwell platform (the Microwell-mesh). Microtissues rapidly accumulate homogenous cartilage-like extracellular matrix (ECM), making them potentially useful building blocks for cartilage defect repair. Cartilage microtissues were cultured for 5 or 10 days and then cryopreserved in 90% serum plus 10% dimethylsulfoxide (DMSO) or commercial serum-free cryopreservation media. Cell viability was maximized during thawing by incremental dilution of serum to reduce oncotic shock, followed by washing and further culture in serum-free medium. When assessed with live/dead viability dyes, thawed microtissues demonstrated high viability but reduced immediate metabolic activity relative to unfrozen control microtissues. To further assess the functionality of the freeze-thawed microtissues, their capacity to amalgamate into a continuous tissue was assess over a 14 day culture. The amalgamation of microtissues cultured for 5 days was superior to those that had been cultured for 10 days. Critically, the capacity of cryopreserved microtissues to amalgamate into a continuous tissue in a subsequent 14-day culture was not compromised, suggesting that cryopreserved microtissues could amalgamate within a cartilage defect site. The quality ECM was superior when amalgamation was performed in a 2% O_2_ atmosphere than a 20% O_2_ atmosphere, suggesting that this process may benefit from the limited oxygen microenvironment within a joint. In summary, cryopreservation of cartilage microtissues is a viable option, and this manipulation can be performed without compromising tissue function.

## Introduction

Cartilage has a limited capacity for spontaneous repair, and defects are more likely to degrade further than regenerate.^1^ This has motivated investment into cell-based therapies, which have already demonstrated modest capacity to repair cartilage defects.^1,2^ A challenge with cell and tissue engineering therapies is balancing efficacy with their high costs,^3^ with cartilage repair therapies having reported costs ranging from $10,000 to $35,000 USD.^4^ Future therapies will look to both improve on current modest efficacies as well as reduce costs.

Matrix-induced autologous chondrocyte implantation (MACI) is the most well-studied cartilage defect repair therapy. In MACI, expanded articular chondrocytes (ACh) are seeded onto a collagen membrane and cultured for few days before being surgically implanted into a cartilage defect site.^5,6^ This requires a Good Manufacturing Practice (GMP) cell manufacturing facility near the clinic and the temporal coordination of the cell manipulation and surgical processes. Orthocell Ltd., an Australian cell therapy company, expands autologous ACh in a GMP facility and then ships frozen cells to clinic.^7^ ACh are thawed in the operating room and directly implanted into the patient. Decoupling surgical and cell manufacturing processes with a cryopreservation step can help to manage capital and procedure costs.

While cryopreservation of a single cell suspension is a standard and reliable process, cryopreservation of larger or more complex tissues is more challenging.^8–11^ During the freezing process, the formation of ice crystals (intra- and extracellular) can cause cellular damage.^12^ In tissues, as water begins to crystallize in the extracellular space, an ionic gradient forms across the cell membrane, causing an efflux of water from inside to outside the cells.^13^ This can lead to dehydration and denaturation of proteins, and cell death.^14,15^ The formation of ice crystals during freezing also subjects the cells to mechanical stress,^2,16,17^ and even in the absence of ice crystal formation, thermal shock during rapid freezing can be sufficient to cause damage.^2^ Controlled rate cooling and cryoprotectant solutions are used to mitigate against these insults.^2,18,19^ Of course, controlling chemical and temperature gradients is more challenging with incrementally larger or thicker tissues.^20^ During the thawing process, cryoprotectants, like dimethylsulfoxide (DMSO) can accumulate at the tissue-solution interface of large tissues, resulting in elevated local concentrations and toxicity.^21^

There is already precedent for the cryopreservation of cartilage or osteochondral tissues. For example, Song et al. reports achieving approximately 80% ACh viability following vitrification of full-thickness femoral heads harvested from rabbits.^22^ Cartilage itself is a theoretically ideal tissue for cryopreservation, as the matrix functions to maintain structure and protect cells during the freeze and thaw process.^1^ There is also precedent for cryopreserving cells that have been aggregated into organoids or microtissues.^10,11,23^ Commercial manufacturers such as STEMCELL™ technologies recommended that their cryopreserved organoids be cultured for a few days and passaged a couple of times after thawing. If the organoids are damaged they will recover during the post-thaw culture.^24,25^ However, need for a post-thaw culture is not compatible with use of cryopreserved organoids or microtissues in cell therapy applications. Instead, it is likely that cell therapy applications of cryopreserved organoids or microtissues will require that the cells in these tissue have a high viability immediately upon thawing.

In previous work, our team developed a high throughput method to manufacture cartilage microtissues from either expanded ACh or bone marrow-derived stromal cells (BMSC).^26,27^ We used a microwell platform, called the Microwell-mesh, to enable the efficient manufacture of hundreds of uniformly sized microtissues. The advantage of microtissues, compared to conventional larger diameter pellet cultures, is that the small diameter reduces metabolite diffusion gradients, yielding more homogenous tissues. Several publications discuss the potential of using microtissues as building blocks in tissue regeneration,^28–30^ and cartilage microtissues may have utility in cartilage defect repair.^31,32^ The suitability of microtissues as an input into cartilage defect repair, may depend on their compatibility with cryopreservation. We hypothesized that their propensity to rapidly accumulate cartilage-like matrix, uniform size and small diameter would make them ideal for cryopreservation.

Herein we optimized the mass production and cryopreservation of cartilage microtissues formed from ACh or BMSC. Microtissues were manufactured in the Microwell-mesh, cultured for 5 or 10 days, and then cryopreserved. After thawing, cell viability and metabolic activity was assessed. Microtissue function was assessed by comparing the capacity of microtissues that had or had not been frozen to amalgamate into a continuous tissue.

## Materials and methods

**Figure 1.**
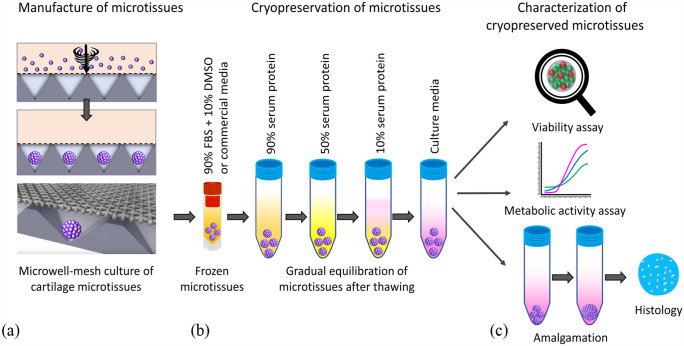
Schematic of the experimental design. (a) ACh or BMSC cells were suspended in chondrogenic culture medium and pelleted into the microwells via centrifugation of the Microwell-mesh plate. Approximately 5000 cells were pelleted into each microwell, yielding microtissues formed from ~5000 cells each. (b) About 40 cartilage microtissues were cryopreserved in 1 mL of cryopreservation solution (90% FBS + 10% DMSO) or CS10 commercial serum-free cryopreservation medium. Microtissues were thawed by gentle swirling the base of the cryotubes in a 37°C water bath and then microtissues slowly washed or equilibrated in descending concentration of FBS or human serum albumin in chondrogenic culture medium, and finally microtissues were transfered into serum-free chondrogenic culture medium used in amalgamation assays. (c) Cryopreserved cartilage microtissues were assessed for viability, metabolic activity, and amalgamation capacity. The thawed microtissues were allowed to amalgamate in chondrogenic culture medium for 14 days and their amalgamation was assessed by histology. Image adapted from Futrega et al.^26,27^

A schematic of the experimental process from manufacture to characterization of cryopreserved cartilage microtissue is depicted in Figure 1. Cartilage microtissues were manufactured from BMSC or ACh and cultured in the Microwell-mesh platform for 5 or 10 days following procedures described previously^26,33^ (Figure 1(a)). Cartilage microtissues were cryopreserved in cryopreservation solution containing 90% fetal bovine serum (FBS; Life Technologies) and 10% dimethylsulfoxide (DMSO) or in CS10 commercial serum-free cryopreservation medium (STEMCELL™ Technologies, Figure 1(b)). After thawing, microtissues were sequentially equilibrated in solutions containing descending concentrations of FBS or human serum albumin in culture medium (Figure 1(b)). Cryopreserved microtissues were characterized for their viability, metabolic activity, and amalgamation capacity (Figure 1(c)). The cryopreserved microtissues were amalgamated for 14 days in serum-free culture medium and amalgamation assessed through histological assays (Figure 1(c)).

### Isolation of human BMSC and expansion

BMSC were isolated from bone marrow aspirates collected from a consenting healthy male (BMSC Donor 1) and female (BMSC Donor 2), both volunteers in their twenties. The collection was performed with ethical approval from the Mater Health Services Human Research Ethics Committee and Queensland University of Technology Human Ethics Committee (Ethics No.: 1300000833). BMSCs were enriched from the donors’ bone marrow by a density gradient separation using Ficoll-Paque (GE Healthcare). Mononuclear cells were suspended in low glucose Dulbecco’s Modified Eagle Medium (LG-DMEM) supplemented with 10% FBS, 100 U/mL penicillin/streptomycin (Pen/Strep; Gibco), 10 ng/mL fibroblast growth factor-1 (FGF-1; Peprotech) and 5 μg/mL porcine heparin sodium salt (Sigma-Aldrich). Cells were cultured overnight in a 20% O_2_ and 5% CO_2_ atmosphere at 37°C as described previously.^26,34^ The next day, the medium and non-adherent cells were aspirated from cultures, and fresh medium added, and the cultures were incubated in a 2% O_2_ and 5% CO_2_ atmosphere at 37°C. When the cells reached ~80% confluency, they were passaged using 0.25% (V/V) Trypsin-EDTA in PBS (Thermo Fisher) and reseeded into new T175 flasks at a density of approximately 1500 cells/cm^2^. BMSCs from the third passage were used in the subsequent studies.

### Isolation of human articular chondrocytes and expansion

Articular cartilage was collected from the knee joint replacement surgery of consenting male patients of age 77 (ACh Donor 1) and 74 (ACh Donor 2) years performed at the St Vincent’s Private Hospital Northside, Queensland, Australia. The collection was conducted with ethics approval granted by the hospital Human Ethics Committee and the Queensland University of Technology (Ethics No. 1400001024). Isolation of chondrocytes from the cartilage was conducted following the previously described protocols.^35^ Briefly, the cartilage tissue was transferred into a petri dish, washed with PBS, and diced into small pieces under aseptic conditions (Supplemental Figure 1). The cartilage tissue slices were digested with Collagenase Type II (300 U/mL) suspended in LG-DMEM supplemented with antibiotic antimycotic solution (0.1 mg/mL streptomycin, 100 U/mL penicillin, and 0.25 μg/mL amphotericin B, Sigma-Aldrich), and incubated overnight at 37°C. The digested tissue suspension was centrifuged at 350*×g* for 5 min. Following centrifugation, the ACh cell pellet was washed three times with LG-DMEM. Isolated ACh were re-suspended in LG-DMEM plus GlutaMAX™ supplemented with 10% FBS, 10 ng/mL FGF-1, and 5 μg/mL porcine heparin sodium salt and the antibiotic antimycotic solution. ACh were expanded in T175 flasks with 35 mL of media and initial cell seeding density of approximately 1500 cells/cm^2^. Cultures were maintained in a 2% O_2_ and 5% CO_2_ atmosphere, at 37°C until becoming ~80% confluent, after which point, they were passaged as described above. ACh were used at passage three in the subsequent studies.

### Microwell-mesh fabrication and preparation

The Microwell-mesh microtissue culture platform consists of an array of microwells, with a nylon mesh bound over the microwell openings. This platform’s fabrication and use was first described by Futrega et al.^26^ The mesh functions to retain microtissues in microwell microwells over extended culture processes. The nylon mesh pore size (36 µm) is big enough to allow single cells to pass through the mesh and into the microwells during cell seeding, but small enough to retain cells once they’ve aggregated into microtissues. The microwell base was fabricated from polydimethylsiloxane (PDMS, SYLGARD™ 184 Silicone Elastomer Kit, Dow Silicones Corporation, Midland, MI, USA) which was cast on a negative microwell patterned polystyrene mold. The microwells had 2 mm × 2 mm square openings and were 0.6 mm deep. Discs were punched from the PDMS sheets using a wad punch (Amazon.com), yielding 3.4 cm diameter discs that fit snuggly into 6-well plates (~250 microwells/disc). A nylon (6/6) mesh with 36 × 36 µm openings was bonded over the microwell opening by applying a thin layer of silicone glue to the top of the microwells and curing at 80°C for 30 min. Excess nylon mesh around the PDMS discs was trimmed and the Microwell-mesh discs were anchored into 6-well plates (Nunc, Thermo Fisher Scientific) with a dab of silicone glue. To sterilize the Microwell-mesh, 4 mL of 80% ethanol was transferred into each well, and the plate centrifuged at 3000×*g* for 5 min. This process displaced air bubbles from the microwells and forced ethanol to contact all surfaces in and under the Microwell-mesh insert. The Microwell-mesh plates were then submerged in 80% (v/v) ethanol bath for 1 h, and then the individual wells were washed thrice with 4 mL of Dulbecco’s phosphate-buffered saline (DPBS) followed by a rinse with water (Invitrogen UltraPure™ DNase/RNase-Free distilled water), dried overnight at 60°C in an oven, and then stored at room temperature until needed. Prior to use in cell culture, the Microwell-mesh were soaked with a Pluronic solution (5% Pluronic-F127 in DPBS w/v; Sigma-Aldrich), which prevents protein absorption onto the PDMS, thus reducing cell attachment, and promoting cell aggregation.^36^ Each well was filled with 4 mL of sterile Pluronic solution, centrifuged 3000×*g* for 5 min forcing the solution into the microwells and displacing bubbles, incubated for 10 min, and then the wells rinsed with PBS, and the wells seeded with BMSC or ACh.

### Manufacturing of cartilage microtissues

BMSCs or ACh were seeded into microwells via forced aggregation. Each well was filled with 3 mL of chondrogenic medium and centrifuged at 3000×*g* at 37°C for 5 min to displace any air bubbles from microwells. Chondrogenic medium was formulated from high glucose DMEM (HG-DMEM) supplemented with 100 U/mL penicillin/streptomycin (Glibco), 100 µg/mL sodium pyruvate (Glibco), 10 ng/mL TGF-β1 (PeproTech), 100 nM dexamethasone (Sigma-Aldrich), 200 mM ascorbic acid 2-phosphate (Sigma-Aldrich), 40 mg/mL L-proline (Sigma-Aldrich) and 1% ITS-X (Gibco). Added to the 3 mL of medium in each well, was 1 mL of cell inoculum containing 1.2 × 10^6^ BMSC or ACh suspended in chondrogenic medium. The cells were pelleted into microwells by centrifuging the plate at 500×*g* for 3 min at 37°C, using forced aggregation to distribute approximately 5000 cells per microwell. Cartilage microtissues were cultured in an incubator with a 2% O_2_ and 5% CO_2_ atmosphere maintained at 37°C for 5 or 10 days, with medium exchange every second day. The aggregation of cells, and the progressive growth of the microtissues was imaged using an Olympus CKX14 microscope, digital camera (Olympus DP26, Japan), and imaging software (CKX14, CellSens Entry).

### Freezing and thawing of cartilage microtissues

The cartilage microtissues were harvested aseptically from the Microwell-mesh as described previously.^27,37^ This was performed by peeling the nylon mesh from the microwells, and then gently using medium aspiration to wash the microtissues into a well plate for collection. Microtissues were suspended in 90% FBS plus 10% DMSO cryogenic solution or cold (4°C on ice) commercial media (CryoStor^®^ CS10, Biolife Solutions, Washington, USA) and aliquoted into Corning 1.2 mL internal threaded polypropylene cryogenic vials. Each vial contained approximately 40 microtissues in 1 mL of cryogenic solution. Microtissues suspended in CS10 were incubated at 4°C for 10 min before freezing as per the manufacturer directions. Vials were transferred to a controlled cooling rate container (Mr. Frosty; Thermo Fisher Scientific), frozen at −80°C for 24 h, and then transferred to liquid nitrogen if stored for greater periods of time. Cartilage microtissues were thawed by gentle swirling the base of the cryotubes in a 37°C water bath. In preliminary studies we observed some cell death when microtissues were transferred from the cryogenic solution to the serum-free chondrogenic medium. In the experiments described here, we first washed away the DMSO containing cryogenic solution with 10 mL medium containing 90% FBS or human serum albumin (2.25 mg/mL in cell culture media) warmed to 37°C, followed by a 5-min soak and wash with 37°C warmed medium containing 50% FBS or human serum albumin (1.25 mg/mL in cell culture media), followed by a 5-min soak and wash with 37°C warmed medium containing 10% FBS or Human serum albumin (0.25 mg/mL in cell culture medium), and finally washed and resuspended microtissues in 37°C warmed serum-free chondrogenic medium. The FBS or human serum albumin culture medium solutions were kept warm at 37°C. Thawed microtissues were gently transferred into 1 mL serum-free chondrogenic culture medium containing tube before allocation for any analysis or amalgamation. Microtissues were imaged before freezing and after thawing to capture any change in dimension as a result of the cryopreservation process.

### Metabolic activity assay of cryopreserved microtissues

The metabolic activity of thawed cartilage microtissues was estimated using the alamarBlue assay. The alamarBlue™ (Thermo Fisher Scientific) solution contains an oxidized non-fluorescent blue dye, Resazurin. Resazurin reacts with the cellular mitochondria and cytoplasm reductase enzymes and is reduced to a fluorescent pink compound, Resorufin.^38^ The change in the fluorescence is commonly used as a surogate measurement of metabolic activity of living cells or tissues.^35,38,39^ The metabolic assay was conducted following the procedure detailed previously.^35,39^ The post-thawed cartilage microtissues were allocated to 24 well plates (Corning Costar TC-treated) in 1 mL of serum-free chondrogenic medium and incubated at 2% O_2_, 5% CO_2_, and 37°C for 4 h to allow for metabolic recovery. Microtissues directly harvested from Microwell-mesh (non-frozen) were used to compare against thawed microtissues. Following incubation, a alamarBlue solution was diluted to 3% in the serum-free chondrogenic culture media of the microtissues. Fluorescence was measured on 544 nm excitation and 590 nm emission from bottom of the plate using a FLUOstar Omega Microplate Reader (BMG LABTECH) at 1, 2, 3, and 4 h. The maximum fluorescence value from the brightest well was used to adjust gain for each well. To enable estimation of the alamarBlue signal per cell, a standard curve of a titration of either BMSC or ACh was performed in parallel with the microtissue metabolic assays.

### Assessment of microtissue viability

To assess microtissue viability, approximately 10 fresh or thawed microtissues from the above alamarBlue assays were transferred to a half-volume 96 well black microplate (Corning). The cartilage microtissues were submerged in PBS containing LIVE/DEAD™ cell viability solution (Thermo Fisher Scientific). Specifically, the solution contained 2 µM calcein-AM (green, stains live cells) and 4 µM of ethidium homodimer-1 (red, stains dead cells) and incubated in dark for 45 min at room temperature. A few microtissues were submerged into 80% ethanol for 2 h to generate dead controls, and then these were then stained with the LIVE/DEAD kit. The stained microtissues were imaged using Olympus IX73 inverted and Olympus FV3000 confocal microscope.

### Amalgamation of cryopreserved microtissues

Fresh or thawed cartilage microtissues were assessed in amalgamation assays. The ACh or BMSC microtissues were allocated to 2 mL sterile microcentrifuge tubes. Each tube contained approximately 40 cartilage microtissues in 1 mL of the serum-free chondrogenic culture medium and were cultured for 14 days. Our previous studies demonstrated that the low oxygen (2% O_2_, 5% CO_2_ concentration) culture of microtissue enhances the matrix production of chondrocytes or chondrogenically induced BMSC.^35,40^ As the amalgamated microtissues would yield a larger tissue, it was not clear if low oxygen or high oxygen cultures would yield superior outcomes. To assess the influence of oxygen, replicate cultures were performed in low oxygen conditions (2% O_2_, 5% CO_2_) or high oxygen conditions (20% O_2_, 5% CO_2_) at 37°C for 14 days with medium exchange every 2 days. Amalgamation characteristics of the cryopreserved cartilage microtissues were compared with the non-frozen microtissues cultured in low or high oxygen conditions.

### Histological assay

Amalgamated microtissues were harvested after 14 days. The amalgamated constructs were washed with PBS, fixed in 4% paraformaldehyde (PFA, Sigma-Aldrich) for 1 h, and embedded in paraffin wax. Embedded cartilage tissues were sectioned using a microtome (RM2235, Leica Biosystems) at 5 µm thickness and collected onto poly-l-lysine coated slides (Thermo Fisher Scientific). Slides were dried in an oven at 60°C for 15 min. Single microtissues were embedded in OCT Tissue-Tek compound (Sakura Finetek) after fixing into 4% PFA for 20 min at room temperature. OCT embedded microtissues were sectioned on a Leica Cryostat CM1950 (Leica Biosystems) and collected at −20°C temperature onto poly-l-lysine coated slides. The prepared slides were stored at −30°C until staining. Paraffin embedded slices were de-paraffinized with xylene and hydrated with a gradient solution of ethanol and water. OCT embedded sections were washed with PBS before processing. Hydrated tissues were acidified with 3% glacial acetic acid (Sigma-Aldrich) in water (v/v) and stained with Alcian blue solution (Sigma-Aldrich) for 30 min at room temperature.^41^ After 30 min, slides were washed thrice with water and counterstained with Nuclear Fast Red (Sigma-Aldrich) for 5 min to identify nuclei in the cartilage tissue sections. The stained slides were dehydrated with gradient solution of ethanol-water solution and cover-slipped using Eukitt quick-hardening mounting medium (Sigma-Aldrich). Stained microtissues were imaged using an Olympus BX63 upright motorized microscope.

### Quantification of glycosaminoglycans (GAG) retention using histological analysis

Alcian blue-stained amalgamated microtissue sections were scanned using Olympus VS120 Slidescanner Microscope (Olympus Corporation, Tokyo, Japan) under a bright-field setting at 40X magnification. The brightness and contrast levels were set similarly for all images. The acquired image of each alcian blue-stained section was converted to a single Tagged image file format (TIFF) such that the entire area of the tissue section was captured. The images were converted to TIFF using Olympus OlyVIA software version 2.9.1. The histological sections were analyzed using open-source software Fiji.^42,43^ A schematic of the analytical process is depicted in Supplemental Figure 2. Briefly, the area of the histological section was traced using *Wand* tool followed by adjusting tolerance at 8-connected mode (Supplemental Figure 2A). The selected area was copied, and outer area of the section was cleared by choosing Edit-clear outside option. Then the images were converted to 8-bit greyscale images (Supplemental Figure 2B). The positive alcian blue-stained area was selected by optimizing each image’s upper and lower threshold value. The threshold values were optimized following selecting a suitable threshold method from the *Auto threshold* function before performing *Threshold* (Supplemental Figure 2C). To calculate the defined positive stain area and intensity, the parameters area fraction and *Mean grey value* were selected at threshold limit (*Limit to threshold*) from the *Analyze-Set measurement* function (Supplemental Figure 2D). The values for the parameters were acquired from *Analyze-measurement* tools (Supplemental Figure 2E). The shift toward a higher mean grey value was proportional to the brightest pixels and considered as reciprocal to alcian blue retention.

### Statistical analysis

Data are presented as mean ± standard deviation (SD) of four biological replicates (*n* = 4). Data analysis was performed using the software GraphPad Prism version 8. Significant differences between groups was determined by one-way analysis of variance (ANOVA) following Tukey’s posthoc test. *p*-value less than 0.05 were considered statistically significant.

## Results

### Microtissue growth and size

**Figure 2.**
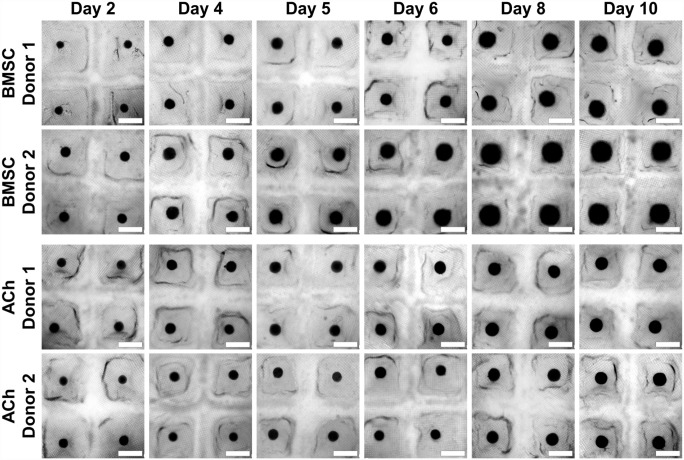
Culture of BMSC and ACh microtissues in the Microwell-mesh. Microtissues in each microwell were formed from approximately 5000 cells. The microtissue culture was continued for 5 or 10 days in chondrogenic medium. Scale bar = 500 µm.

**Figure 3.**
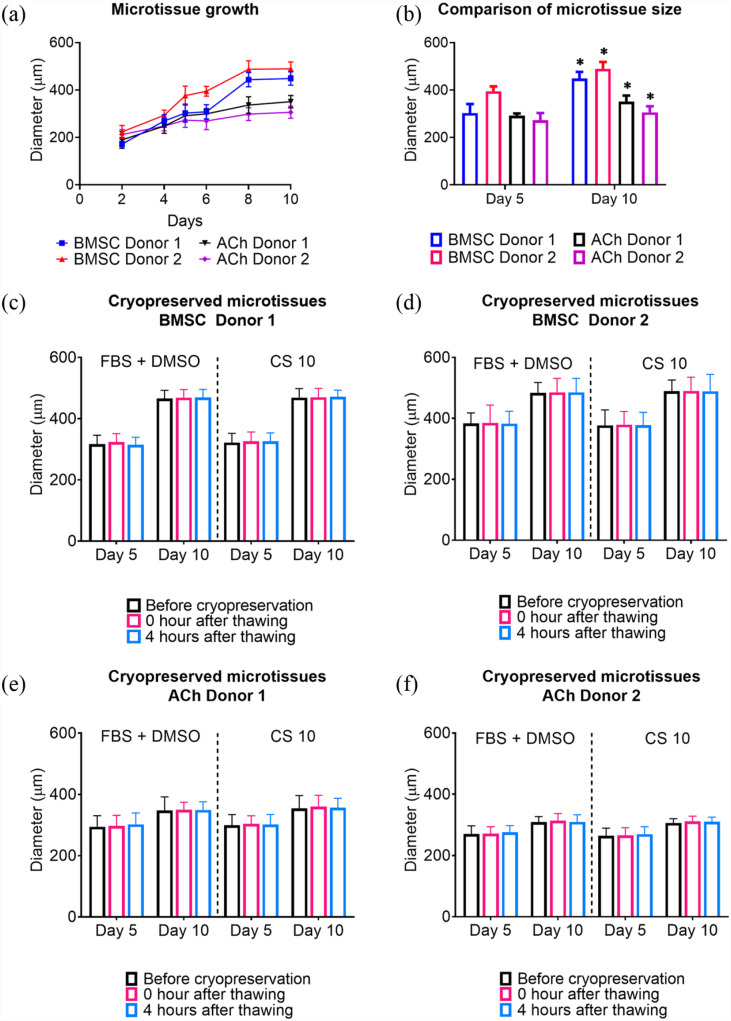
Comparison of BMSC and ACh derived microtissue size grown for 5–10 days. (a) represents the diameter of the microtissues measured every 2 days and before harvesting from microwell-mesh. (b) represents comparison of size between 5 and 10 days microtissue fromed from BMSC or ACh. (c and d) show the size comparison of BMSC Donor 1 and Donor 2 derived cryopreserved microtissues before freezing and immediately (0 h) and 4 h after thawing into culture media. (e and f) represent the size comparison of BMSC derived cryopreserved ACh Donor 1 and Donor 2 microtissues before freezing and immediately (0 h) and 4 h after thawing into culture media. Data are presented as mean ± SD (*n* = 16). * Represents *p* < 0.05 compared to 5 days cultured microtissues. As shown in (c) to (f) cryopreservation did not result in a statistically significant difference in the diameter of non-cryopreserved and cryopreserved microtissues.

BMSC or ACh self-assembled into microtissues in the Microwell-mesh. As depicted in Figure 2, BMSC and ACh microtissue progressively increased in size over the 5 or 10 days culture period, similar to our previous observations^26,27^ The diameter of microtissues was tracked every 2 days before changing the culture media and harvesting. Figure 3(a) shows that BMSC microtissue size was greater than ACh microtissues. BMSC microtissues increased in size until day 6 or 8 of culture, after which relative increase in size slowed. By contrast, there was a more gradual increase ACh microtissue size over the culture period. ACh or BMSC microtissues cultured for 10 days were significantly larger than microtissues cultured for 5 days (Figure 3(b)). Any alteration in size due to the cryopreservation was evaluated by measuring the microtissue diameter before freezing and after thawing. As delineated in Figure 3(c) to (f), cryopreservation did not alter microtissue diameter, and the microtissue diameter remained constant in the 4 h of incubation into the culture medium after thawing. Non-cryopreserved BMSC and ACh microtissues also showed similar characteristics after harvesting from the microwell-mesh platform (Supplemental Figures 3A–D). The results also demonstrated that there was not any detectable difference in diameter between the microtissues cryopreserved in 90% FBS + 10% DMSO and those cryopreserved in CS10.

### Microtissue viability

The viability of cells in non-cryopreserved and cryopreserved cartilage microtissues was assessed using viability dyes. Early in the optimization process, microtissues frozen in the cryogenic solutions were thawed into serum-free chondrogenic medium, rather than incrementally in decreasing serum concentrations. When tissues were immediately transitioned to serum-free medium this resulted in many non-viable cells (Supplemental Figure 4A), and we presume that this cell death was caused by oncotic shock.^2,44,45^ The non-viable cells at the surface of the microtissues appeared to contribute to a layer of abnormal tissue and ECM at the interface of microtissues in the amalgamated tissue (Supplemental Figure 4B). The formation of this undesirable layer of tissue appears to be indicative of cell death at the surface of the microtissues and provided impetuous for the development a new method for microtissue cryopreservation where the goal was to improve post-thaw viability. In this modified protocol, after thawing the microtissues cryopreserved in 90% FBS + 10% DMSO or CS 10 microtissues were transferred into medium supplemented with 90% FBS or human serum albumin (2.25 mg/mL in cell culture media). This first step was intended to eliminate the DMSO, but maintain the high protein content the cells had been exposed to in cryopreservation medias. Then, the concentration of the serum was gradually diluted in 50% FBS or 1.25 mg/mL human serum albumin, 10% FBS or 0.25 mg/mL human serum albumin in chondrogenic medium before amalgamation of the BMSC or ACh microtissue tissues in serum-free chondrogenic medium.

**Figure 4.**
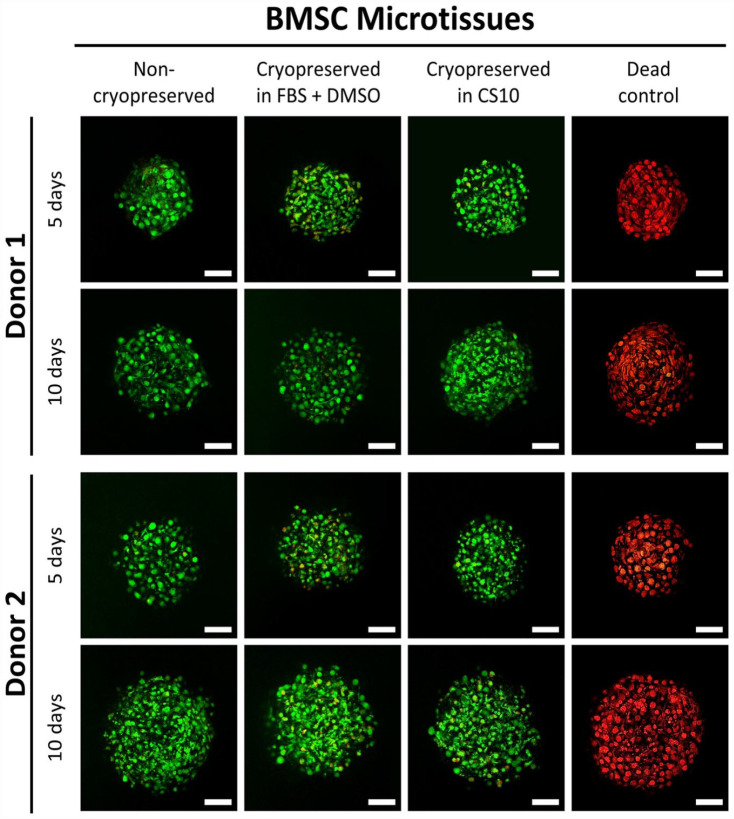
Viability of BMSC microtissues. Microtissues grown from BMSC for 5 or 10 days were cryopreserved and stained with calcein-AM (viable, green) and ethidium homodimer-1 (non-viable, red). Column images with “Dead” caption represent microtissue that had been submerged in 80% ethanol for 2 h prior to staining, and these served as a dead cell control. Scale bar = 50 µm.

**Figure 5.**
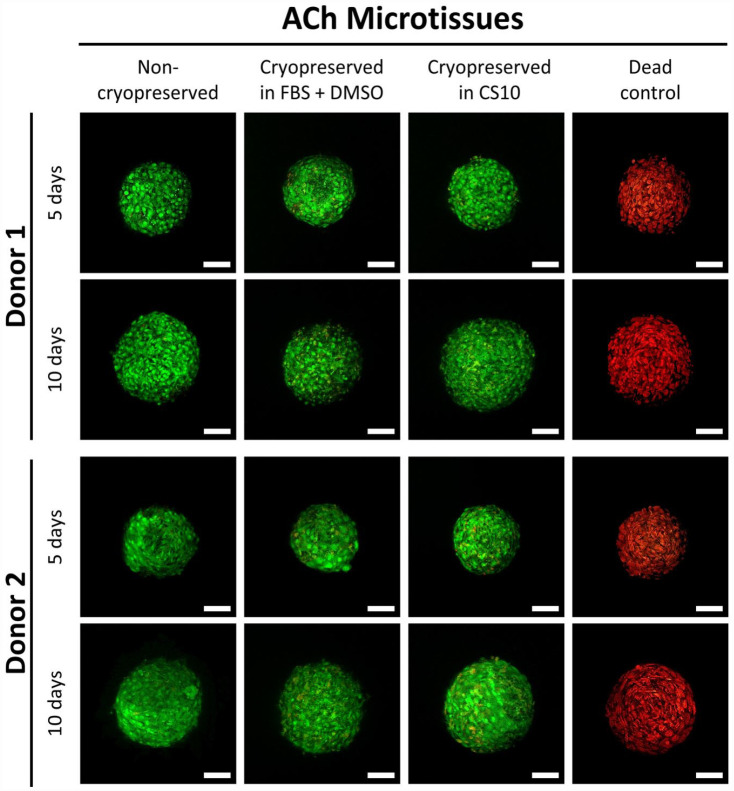
Viability of ACh microtissues. Microtissues grown from ACh for 5 or 10 days were cryopreserved and stained with calcein-AM (viable, green) and ethidium homodimer-1 (non-viable, red). Column images with “Dead” caption represent microtissue that had been submerged in 80% ethanol for 2 h prior to staining, and these served as a dead cell control. Scale bar = 50 µm.

Using the improved cryopreservation method, which included thaw into incremental dilutions of FBS or medium supplemented with human serum albumin, fresh and thawed microtissues were compared once again. Non-viable cells were rare in fresh BMSC or ACh microtissues as shown in Supplemental Figure 5. Supplemental Figure 5 shows multiple microtissues captured in projection images. To capture more detail, and to look for non-viable cells, we used confocal microscopy (Figures 4 and 5). Similarly, confocal microscopy revealed nearly 100% cell viability in both non-cryopreserved and cryopreserved cartilage microtissues. Because dead cells were so uncommon, we included a parallel dead cell control where the microtissues had been submerged in 80% ethanol for 2 h to induce cell death, and all of the cells in these microtissues stained with the ethidium homodimer-1 (red, indicating dead cells), and not with the calcein-AM (green, indicating live cells).

### Metabolic activity

**Figure 6.**
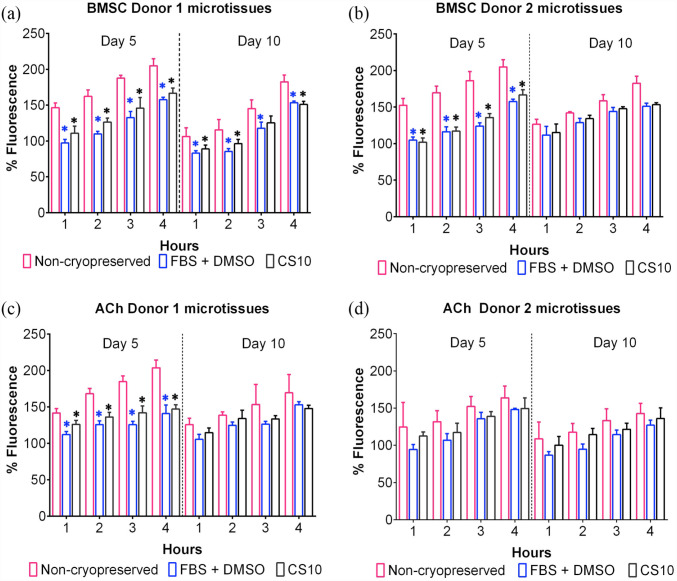
Metabolic activity of cryopreserved and non-cryopreserved microtissues. Data shown left of the dotted line represents the metabolic activity of 5-day BMSC or ACh microtissue and right shows for 10-day microtissues. (a and b) shows the difference of metabolic activity between cryopreserved and non-cryopreserved microtissues derived from BMSC Donor 1 and BMSC Donor 2. (c and d) shows the difference of metabolic activity between cryopreserved and non-cryopreserved microtissues derived from ACh Donor 1 and ACh Donor 2. Data are presented as mean ± SD (*n* = 4). *Represents *p* < 0.05 compared to non-cryopreserved microtissues.

As shown by Figure 6, the metabolic conversion of alamarBlue of both cryopreserved and non-cryopreserved cartilage microtissues increased as the culture was extended. Five day old non-cryopreserved BMSC Donor 1 (Figure 6(a)), BMSC Donor 2 (Figure 6(b)), and ACh Donor 1 (Figure 6(c)) microtissues demonstrated significantly greater metabolic activity compared to their cryopreserved counterparts. The metabolic function of 10 day cultured BMSC Donor 2 (Figure 6(b)), ACh Donor 1 (Figure 6(c)), and ACh Donor 2 (Figure 6(d)) derived cryopreserved microtissues were found to be almost identical to non-cryopreserved microtissues. The metabolic activity of the ACh or BMSC microtissues grown for 5 days was noticeably higher than the 10 days old microtissues in both the cryopreserved and non-cryopreserved conditions (Figure 4, Supplemental Figure 2). The alamarBlue fluorescence was linear when plotted against the different concentrations of BMSC and ACh Donor cells from 1 to 4 h (Supplemental Figures 6B and 7A–D). Metabolic activity of microtissues appeared to be only marginally compromised by cryopreservation when they were incrementally exposed to decreasing serum concentrations during thawing. Otherwise, metabolic activity was significantly compromised for the microtissue cryopreserved either in FBS + DMSO or commercial cryopreservation solution, CS10 (Supplemental Figure 6A).

### Histology and amalgamation of cryopreserved microtissue

**Figure 7.**
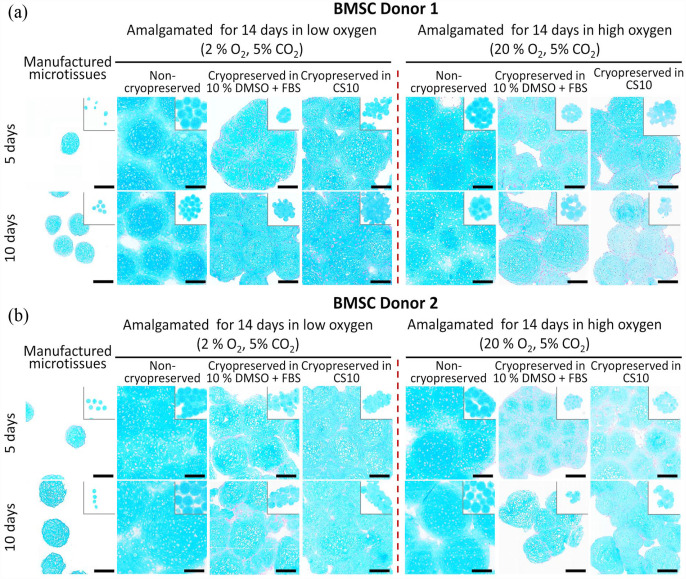
Alcian blue staining of cryopreserved and non-cryopreserved amalgamated BMSC derived cartilage microtissues in different oxygen conditions. BMSC derived microtissues were cultured for 5 or 10 days and frozen in cryopreservation medium. Non-cryopreserved and cryopreserved microtissues were amalgamated in low oxygen (2% O_2_, 5% CO_2_) or high oxygen conditions (20% O_2_, 5% CO_2_) at 37°C conditions for 14 days. (a and b) represents Alcian blue staining of BMSC Donor 1 and Donor 2 non-cryopreserved and cryopreserved amalgamated microtissues in different oxygen conditions. Scale bar = 100 µm.

**Figure 8.**
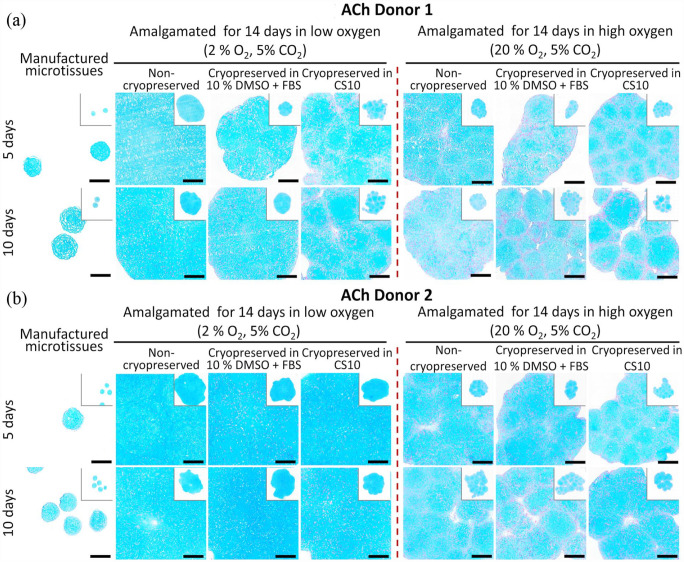
Alcian blue staining of cryopreserved and non-cryopreserved amalgamated ACh derived cartilage microtissues in different oxygen conditions. ACh derived microtissues were cultured for 5 or 10 days and frozen in cryopreservation medium. Non-cryopreserved and cryopreserved microtissues were amalgamated in low oxygen (2% O_2_, 5% CO_2_) or high oxygen conditions (20% O_2_, 5% CO_2_) at 37°C conditions for 14 days. (a and b) represents Alcian blue staining of ACh Donor 1 and Donor 2 non-cryopreserved and cryopreserved amalgamated tissues in different oxygen conditions. Scale bar = 100 µm.

Cryopreserved microtissues matured for 5 or 10 days in the Microwell-mesh platform were thawed and washed using gradient series of FBS-supplemented chondrogenic medium. The microtissues were amalgamated for further 14 days, and histological sections of the amalgamated tissues stained with Alcian blue (Figure 7). Cryopreserved microtissues amalgamated in low oxygen (2% O_2_, 5% CO_2_) condition retained intense and evenly distributed Alcian blue staining (Figures 7(a), (b), 8(a) and (b)). These data suggest that despite the greater size of the amalgamated tissues, low oxygen led to superior matrix accumulation for both amalgamated ACh and BMSC microtissues. While matrix production may have been inhibited by higher oxygen, this did not necessarily equate to poor integration. Thus, it is possible that integration and matrix production are at least partially decoupled. Non-cryopreserved BMSC microtissues had greater Alcian blue staining, relative to cryopreserved microtissues (Figures 7(a), (b), 8(a) and (b)) in both the low oxygen (2% O_2_, 5% CO_2_) and high oxygen (20% O_2_, 5% CO_2_) conditions. Cryopreserved ACh microtissues (Figure 8(a) and (b)) better tolerated the freeze-thaw process relative to BMSC microtissues in both conditions (Figure 7(a) and (b)), and cryopreserved day 5 ACh microtissues for Donors 1 and 2 (Figure 7(a) and (b)) achieved nearly seamless amalgamation in the low oxygen conditions. Alcian blue staining by the amalgamated tissue formed from the cryopreserved 5 and days 10 day ACh microtissues was almost identical to the non-cryopreserved amalgamated microtissues (Figure 7(a) and (b)). By contrast to the data in Figure 8, when thawed microtissues were immediately transferred to serum-free chondrogenic medium, GAG deposition as assessed through Alcian blue staining was compromised (Supplemental Figure 4B).

### Glycosaminoglycans (GAG) retention

**Figure 9.**
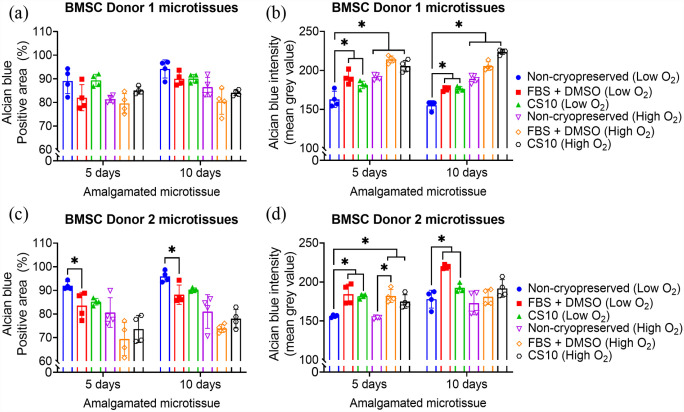
Quantification of GAG from Alcian blue-stained amalgamated BMSC microtissues. BMSC microtissues were cultured for 5 or 10 days and cryopreserved in 90% FBS + 10% DMSO or CS10. Cryopreserved BMSC microtissues were thawed and allowed to amalgamate for further 14 days in low oxygen (2% O_2_, 5% CO_2_) or high oxygen (20% O_2_, 5% CO_2_) conditions at 37 C. (a-d) represents percentage of positive Alcian blue stained area of BMSC Donor 1 and Donor 2 non-cryopreserved and cryopreserved microtissues amalgamated in different oxygen conditions. Data are presented as mean ± SD of four replicates. * Represents *p* < 0.05 compared to non-cryopreserved microtissues.

**Figure 10.**
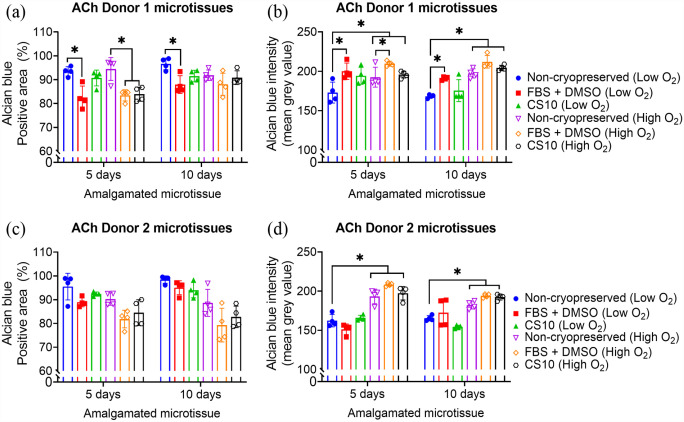
Quantification of GAG from Alcian blue stained ACh amalgamated microtissues. ACh microtissues were cultured for 5 or 10 days and cryopreserved in 90% FBS + 10% DMSO or CS10. Cryopreserved ACh microtissues were thawed and allowed to amalgamate for further 14 days in low oxygen (2% O_2_, 5% CO_2_) or high oxygen (20% O_2_, 5% CO_2_) conditions at 37°C. (a-d) represents percentage of positive Alcian blue stained area of ACh Donor 1 and Donor 2 non-cryopreserved and cryopreserved microtissues amalgamated in different oxygen conditions. Data are presented as mean ± SD of four replicates. * Represents *p* < 0.05 compared to non-cryopreserved microtissues.

BMSC and ACh microtissue cultured for 10 days showed significantly higher GAG deposition, as estimated by alcian blue intensity, compared to micrtissutes cultured for 5 days (Supplemental Figure 9). As depicted in Figures 9, 10(a) and (c), both 5 and 10 days matured BMSC and ACh-derived microtissues cryopreserved in CS10 retained a similar percent of Alcian blue positive area compared to non-cryopreserved microtissues amalgamated in a low oxygen atmosphere. By contrast, the percentage of Alcian blue retention was significantly reduced for both non-cryopreserved and cryopreserved microtissues amalgamated in a high oxygen atmosphere (Figures 9 and 10). The mean grey value between the ACh Donor 2 derived non-cryopreserved and cryopreserved amalgamated microtissue was similar, indicating an insignificant difference of Alcian blue retention when using low oxygen culture conditions (Figure 10(c)). However, the GAG retention for the ACh Donor 2 derived cryopreserved microtissues was significantly reduced in high oxygen culture conditions (Figure 10(d)). On the other hand, cryopreservation of 5 days and 10 days ACh Donor 1 microtissues in CS10 did not significantly alter the GAGs deposition or Alcian blue retention, compared to non-cryopreserved amalgamated microtissues (Figure 10(a) and (b)). The intensity of Alcian blue remained unaffected following cryopreservation of 10 days matured ACh Donor 2 microtissues in FBS + DMSO (Figure 10(a)). A significant reduction in GAGs deposition, as estimated by means of Alcian blue intensity, was observed when BMSC Donor 1 or BMSC Donor 2 derived microtissues were amalgamated in high oxygen culture conditions (Figure 9(b) and (d)). GAGs deposition, as estimated by means of Alcian blue intensity, was found to be similar for non-cryopreserved and cryopreserved microtissues amalgamated in low oxygen conditions (Figure 9(a) and (c)). The GAGs production was not significantly different between the FBS + DMSO and CS10 cryopreserved microtissues amalgamated in low oxygen or high oxygen conditions (Figures 9 and 10). GAG production was significantly diminished for ACh-derived microtissue that didn’t undergo sequential dilution described in Figure 1 after thawing from FBS + DMSO or CS10 cryopreservation solution (Supplemental Figure 8).

## Discussion

Several cell and biomaterial strategies are being explored in an effort to repair or extend the functional life of articular cartilage. Biomaterial-based methods include strategies that aim to directly replace damaged articular cartilage with material, or establish a biomaterial-cell composite, or extend function by providing additional joint lubrication (reviewed here^46^ and here^47^). A major consideration in the development of cell-based therapies is the compatibility of the cell manufacturing processes with scaling and cryopreservation. Microtissue cultures are often used because the small diameter and uniform tissue size reduces diffusion gradients, making it possible to maintain high cell viably across relatively complex tissues.^26,48^ In the cartilage tissue engineering field, microtissues have proven useful for generating cartilage-like tissue with relatively uniform ECM,^26,27,40^ and have demonstrated utility as building blocks for generating larger tissues that could be used for cartilage defect repair.^31,32,35^ We reasoned that the same small diameter and minimal gradient features that lead to relatively uniform cartilage matrix in microtissues would also make microtissues compatible with cryopreservation. Herein, we used a high throughput microwell platform to generate hundreds of cartilage microtissues from BMSC or ACh, cryopreserved these microtissues, and then assessed cell viability, metabolic activity, and the capacity of the thawed microtissues to amalgamate into a continuous tissue in assays meant to test the minimum function requirements necessary to repair a cartilage defect.

Microtissues assembled from BMSC or ACh rapidly increased in diameter, and this increase in tissue size is largely associated with the accumulation of cartilage-like ECM. In previous work, our group has shown that microtissue size is driven mainly by an increase in ECM accumulation, with relatively modest increases in cell number (using DNA as a surrogate measure of cell number).^26,27,49^ In the previous study which most closely replicates the one described here,^26^ we found no statistical difference in the DNA content of BMSC microtissues cultured for 7 or 14 days. A benefit of the accumulation of ECM is that the matrix imparts mechanical support onto the microtissue, functioning to physically protect cells during the cryopreservation and cell manipulation processes. In previous work, using cartilage microtissues that had not been cryopreserved, we observed that the capacity of the microtissues to amalgamate declined as the microtissue matured and matrix accumulated.^32^ For example, we observed that amalgamation was compromised when microtissues had first been cultured for greater than 10 days as discrete microtissues before initiating the amalgamation process.^32^ Thus, while extending the culture contributes to further matrix accumulation, this accumulated matrix eventually compromises the capacity of microtissues to amalgamate. Based on these data, we used microtissues that had been cultured for 5 or 10 days in the cryopreservation studies described here.

BMSC or ACh microtissues that had been culture for 5 or 10 days tolerated cryopreservation well, and very few non-viable cells were detected after thawing. It is common in research laboratories to freeze BMSC or ACh in 90% FBS + 10% DMSO.^26,27,40^ In preliminary work, we transferred microtissues thawed from a 90% FBS + 10% DMSO solution directly into serum-free chondrogenic medium. While cell viability was relatively high, we did observe some dead cells on the surfaces of these microtissues. When these microtissues amalgamated there was a layer of ECM that formed between microtissues that did not remodel and stain weakly with Alcian blue. We assumed that this layer formed from dead cells at the surface of microtissues which had been trapped in the ECM. We sought to improve the thawing process by gradually transitioning the tissues from serum-rich cryopreservation medium to serum-free chondrogenic culture medium. By incrementally diluting the serum concentration from 90% to 50% to 10%, and finally to serum-free, cell viability after thawing was virtually 100% for both BMSC and ACh microtissues, and so this method was used in all subsequent studies. To mimic this dilution series in an animal product free formulation, we used medium supplemented with 2.25, 1.25, and 0.25 mg/mL human serum albumin. It is well known that the cryopreservation of stem cells can be improved through the use of serum in the cryopreservation medium.^50,51^ It is thought that the protein in the serum, specifically the high concentration of albumin, functions to balance the colloid oncotic pressure across the sensitive cell membrane during the freeze and thaw process.^52,53^ Of course, the sudden transition from protein-rich cryopreservation medium to protein-replete chondrogenic medium likely causes oncotic pressure gradients sufficient to rupture some cell membranes.^54–57^ In assays where high BMSC or ACh viability is required immediately after thawing, we recommend that researchers consider an incremental transition from high protein content freezing medium to lower protein content culture medium.

Using optimized thawing, ACh and BMSC microtissue viability was high, although metabolic activity was reduced compared to non-cryopreserved microtissues. Previous studies have demonstrated that cell metabolism can change from aerobic to anaerobic following cryopreservation.^58^ Because Alamar blue uses NADPH dehydrogenase or NADH dehydrogenase enzyme of the aerobic respiratory pathway,^59^ a change from aerobic to anaerobic respiration would suggest a down regulation of metabolic activity, rather than a switch in metabolic activity. It is reasonable to envision that cells may either have compromised respiration or may use less efficient metabolic processes immediately following cryopreservation, and for this reason, metabolic assays should be used in combination with viability staining; do not rely on a single assay to assess viability. Future studies might use tools such as Agilent’s Seahorse XF Pro to quantify both metabolic activity as well as which metabolic pathways are being utilized before and after cryopreservation. Understanding if there is truly reduced metabolic activity or a switch in metabolic pathways will enable understanding if there is damage inflicted by the cryopreservation process, and possibly how to prevent it.

More important than viability staining or metabolic activity, is assessing microtissue function. Following cryopreservation, both BMSC and ACh microtissues demonstrated excellent amalgamation capacity. ACh microtissues appeared effectively unchanged by cryopreservation, while BMSC microtissues appeared to be only modestly compromised. In both cases, microtissues that had been cultured for only 5 days prior to amalgamation performed better than those that had been cultured for 10 days prior to amalgamation. In previous studies, our team has demonstrated that cartilage microtissues benefit by being culture in low oxygen atmospheres (2% O_2_) compared to high oxygen atmospheres (20% O_2_).^26,35,37,40^ Because amalgamated tissues are bigger than single microtissues, we were uncertain if these tissues would benefit from low oxygen or high oxygen conditions. The benefits of low oxygen remained profound. While microtissues did amalgamate in high oxygen conditions, the matrix that formed the bond between microtissues stained weakly with Alcian blue. A potential positive of this behavior is that the low oxygen environment in a joint may function to enhance the amalgamation of microtissues. Evaluation of both fresh and cryopreserved microtissues in a small or large animal model should be performed to understand how the process may impact long-term cell fate and tissue function.

Cryopreservation of cartilage microtissues is straight-forward, although high cell viability requires a gradual transition to serum-free medium following thawing, and amalgamation significantly benefits from being performed in a low oxygen atmosphere. It is possible that cartilage microtissues or other cartilage tissue cryopreservation efforts failed, and are unreported, simply because one of these two critical conditions was not met. The capacity to easily cryopreserve cartilage microtissues may increase their practical utility in clinical cartilage defect repair applications. Microwell platforms, like the Microwell-mesh, already enable efficient high throughput manufacture of microtissues. Microtissues, more so than large diameter pellets, are likely to pack well into cartilage defect sites, and their small diameter makes them well suited to minimally invasive arthroscopic delivery. We envision a future where frozen microtissues might be delivered to clinics and effectively injected into defect sites using minimally invasive arthroscopic tools. The next major innovation would be the development of methods to produce chondrocyte-like cells from pluripotent cell populations suitable for use as universal donor cells. A number of groups are working to generate universal donor cell lines that could be used in off the shelf therapies.^60^ Coupling of these cell lines, with high throughput microtissue manufacturing and cryopreservation protocols, like the one described here, could offer a pathway for financially viable cartilage defect repair.

## Supplemental Material

sj-docx-1-tej-10.1177_20417314231176901 – Supplemental material for Method for manufacture and cryopreservation of cartilage microtissues Click here for additional data file. Supplemental material, sj-docx-1-tej-10.1177_20417314231176901 for Method for manufacture and cryopreservation of cartilage microtissues by Md. Shafiullah Shajib, Kathryn Futrega, Rose Ann G Franco, Eamonn McKenna, Bianca Guillesser, Travis J Klein, Ross W Crawford and Michael R Doran in Journal of Tissue Engineering
