# Supplementary material for: Method for manufacture and cryopreservation of cartilage microtissues
Source: J Tissue Eng. 2023 Jul 26;14:20417314231176901. doi: 10.1177/20417314231176901 (PMC10387698; doi:10.1177/20417314231176901)
Supplement: sj-docx-1-tej-10.1177_20417314231176901 – Supplemental material for Method for manufacture and cryopreservation of cartilage microtissues [file sj-docx-1-tej-10.1177_20417314231176901.docx]

# Supplementary Materials

# Method for manufacture and cryopreservation of cartilage microtissues

Md. Shafiullah Shajib, Kathryn Futrega, Rose Ann G. Franco, Eamonn McKenna, Bianca Guillesser, Travis J. Klein, Ross W. Crawford, Michael R. Doran


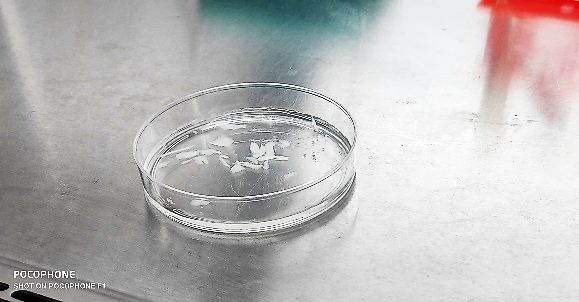

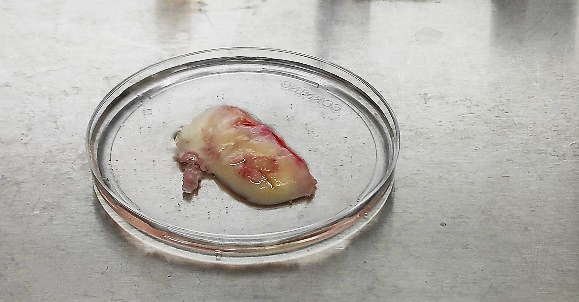

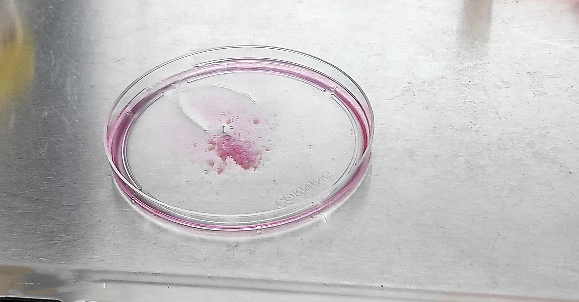

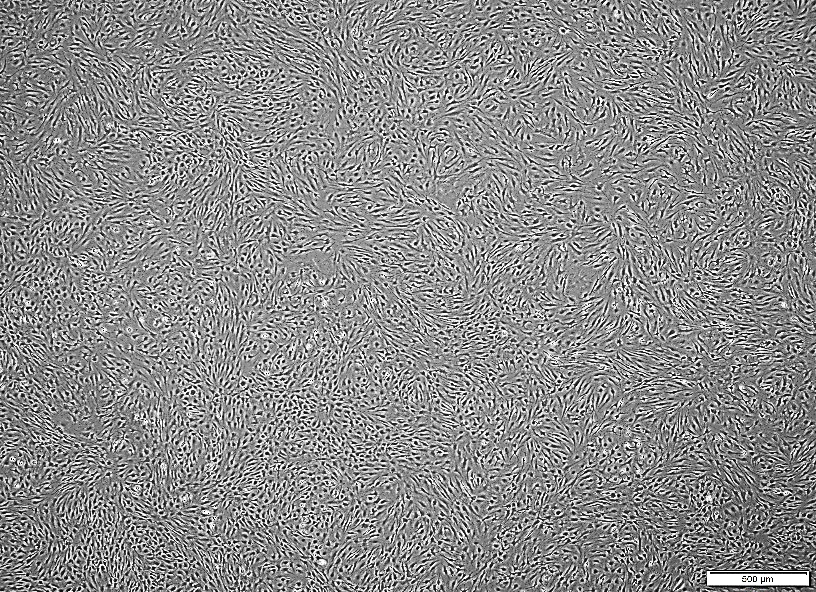


**(A)**

**(B)**

**(C)**

**(D)**

**Supplementary Figure 1**: Isolation of chondrocytes from surgical discards collected from joint replacement surgery. **(A)** Collected cartilage piece was washed with PBS to remove any blood and other surgical waste. **(B)** The cartilage portion was kept moist with sterile PBS to avoid drying off in a petri dish. The surgical tissue portion was carefully excised from its surface to get pieces of thin glossy white cartilage tissue using sterile scalpels and forceps. **(C)**  Cartilage chunks were chopped into small pieces using scalpels in a new petri dish supplied with 1 mL of collagenase type II enzyme digestion solution. (**D**) Isolated ACh were expanded to reach confluency (image scale = 500 µm).

**Supplementary Figure 2**: Quantification of GAG by means of alcian blue intensity. Step **(A)** shows the area of the section was selected using wand tool. The tolerance value was optimized for each image at 8-connected mode. Step **(B)** shows the outer area of the image was cleared by using Edit🡪 clear outside function. Then the RGB image was converted to 8 bit image selecting Image 🡪 Type 🡪 8 bit options. Step **(C)** demonstrates the positive stained area was selected by using threshold function. The upper and lower threshold value was optimized from a pre-defined method from Auto threshold function. In this image the value for MaxEntropy method was found to be suitable. The minimum and maximum threshold values were set and the alcian blue stained area was observed in red colour. **Step (D)** shows the parameters selected for calculating the area of positive stain, the Area, Area fraction, Mean grey value and Limit to threshold parameter and those were set by selecting Analyze 🡪 Set measurement function. **Step (E)** show the generating the results for the selected parameters which were obtained by selecting Analyze 🡪 measurement option.


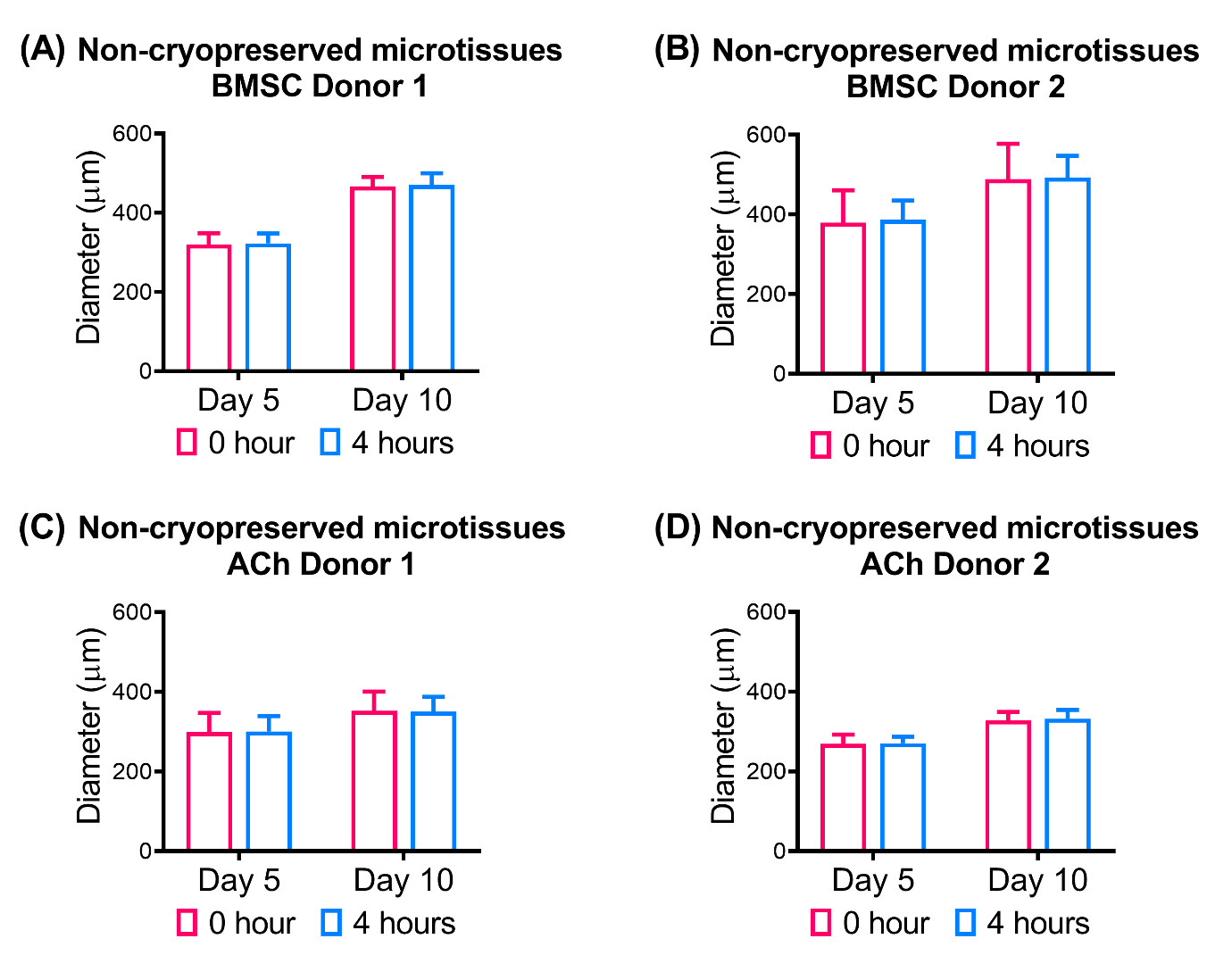


**Supplementary Figure 3**: Comparison of non-cryopreserved BMSC and ACh derived microtissue size grown for 5-10 days. **(A)** and **(B)** represent the diameter difference between 5 and 10 days matured microtissues of BMSC Donor 1 and BMSC Donor 2 immediately (0 hours) after harvesting from microwell-mesh and after 4 hours of incubation into culture media. **(C)** and **(D)** represent the diameter difference between 5 and 10 days matured microtissues from ACh Donor 1 and ACh Donor 2 population immediately (0 hours) after harvesting from microwell-mesh and after 4 hours of incubation into culture media. Data are presented as mean ± SD (n = 16). * represents p < 0.05 compared to 5 days matured microtissues.


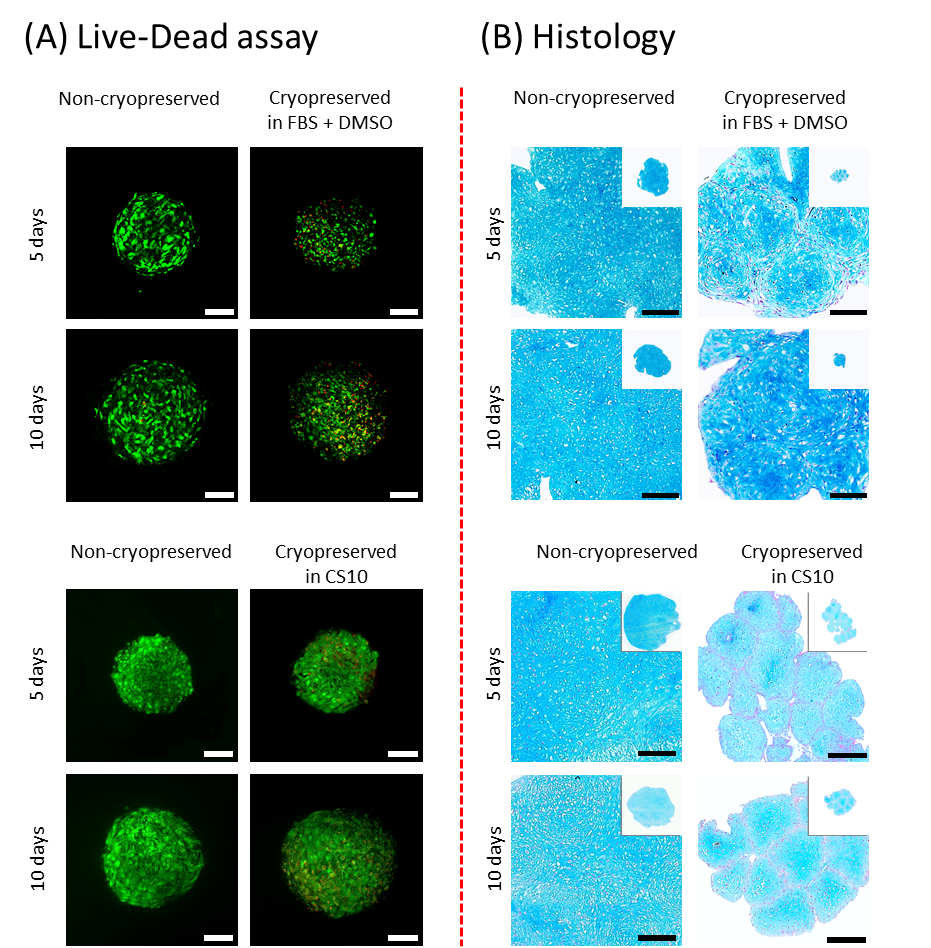


**Supplementary Figure 4**: Viability and amalgamation of articular chondrocytes (ACh) donor 2 derived 5 days and 10 days matured cryopreserved and non-cryopreserved cartilage microtissue. Cryopreserved microtissues were frozen with 10% DMSO in FBS. Non-cryopreserved microtissues were used directly from microwell-meshed without freezing. (A) represents Live/Dead image of the cryopreserved and non-cryopreserved microtissues. Images scale bar = 50 µm. (B) represents histological sections of amalgamated cryopreserved and non-cryopreserved microtissues stained with alcian blue. Images scale bar = 100 µm.


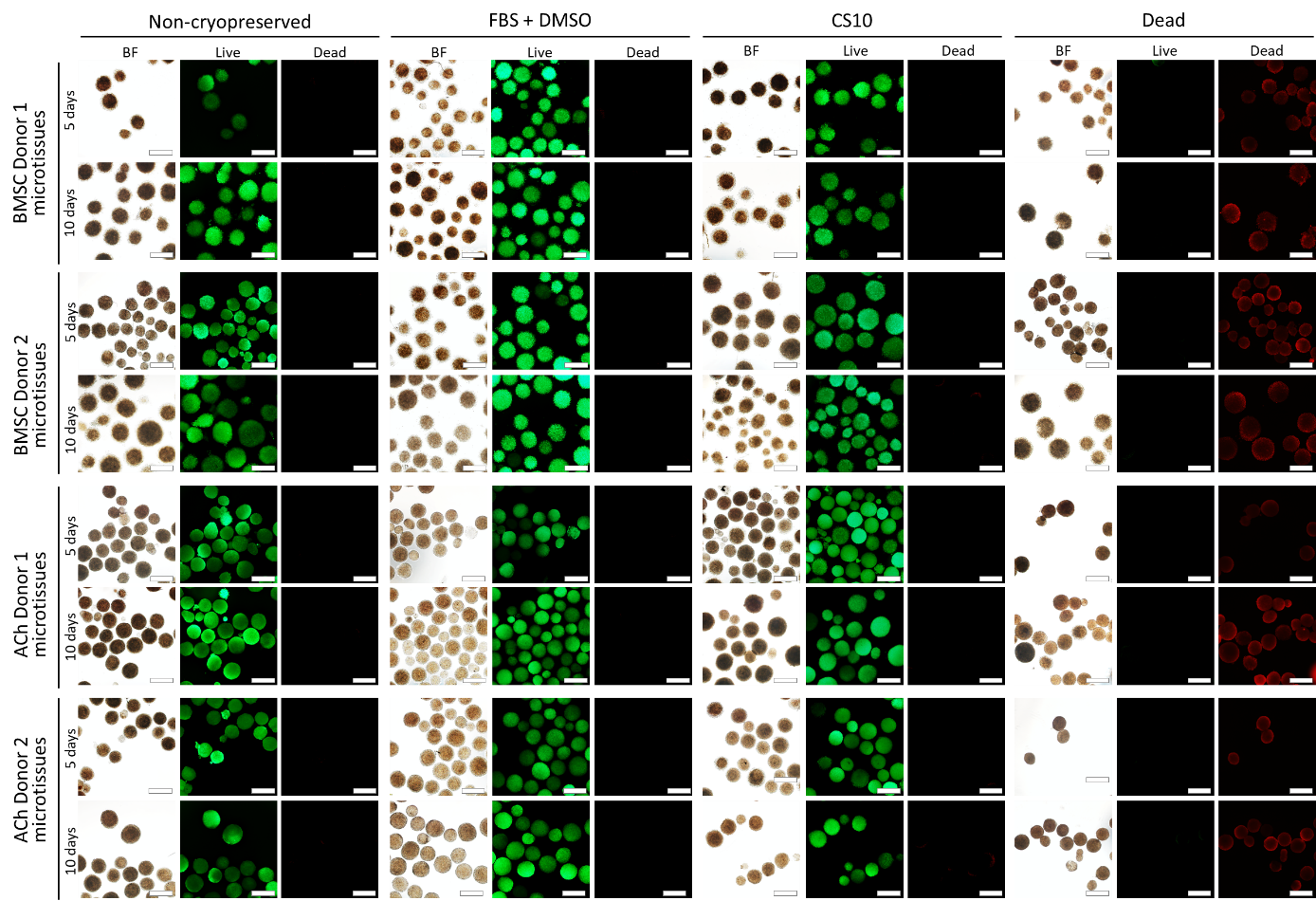


**Supplementary Figure 5**: Live/Dead staining of cryopreserved and non-cryopreserved cartilage microtissue derived from BMSC and ACh. BF= Bright field images of microtissues. “Live” and “Dead” represents microtissues imaged for live cells (green fluorescent) and dead cells (red) following simultaneous staining with calcein-AM and ethidium homodimer-1 (red fluorescent). Dead (control) group represents the dead cartilage microtissue which were submerged into 80% ethanol for 1 hour. Image scale bars = 200 µm.


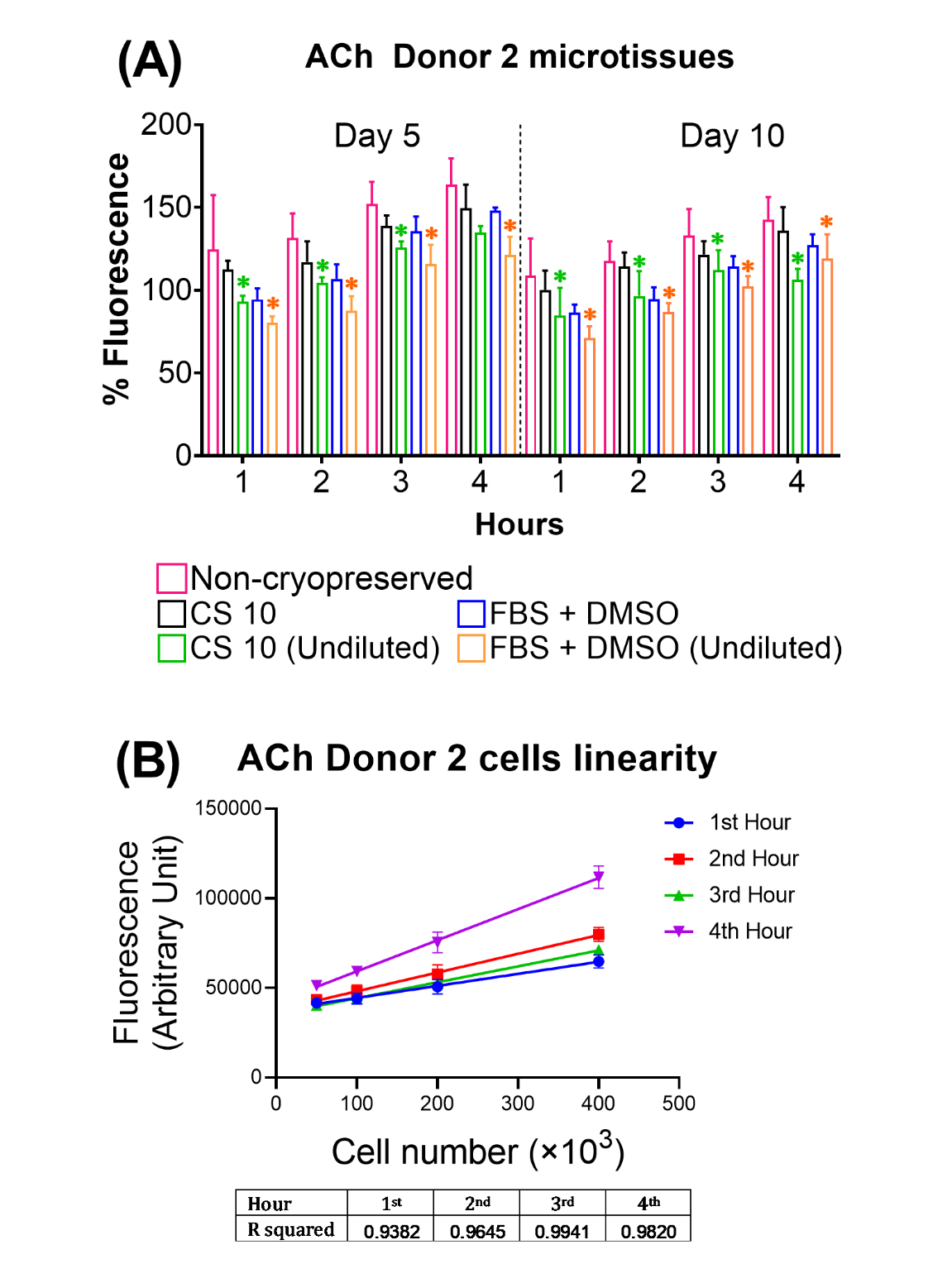


**Supplementary Figure 6**: Metabolic activity of Aritcular chondrocytes (ACh) donor 2 derived microtissues or cells in AlamarBlue assay. (A) represents difference of metabolic activity between cryopreserved (in 10% DMSO in FBS and did not undergo step wise dilution with FBS and medium after thawing) and non-cryopreserved microtissues. Data shown left of the dotted line represents the metabolic activity of 5 days matured microtissue and right shows for 10 days matured cryopreserved and non-cryopreserved microtissues. (B) represents linear curves of the ACh donor 2 derived cells in AlamarBlue assay at different time point. Data are presented as mean ± SD (n = 4). * represents *p* < 0.05 compared to cryopreserved microtissues.


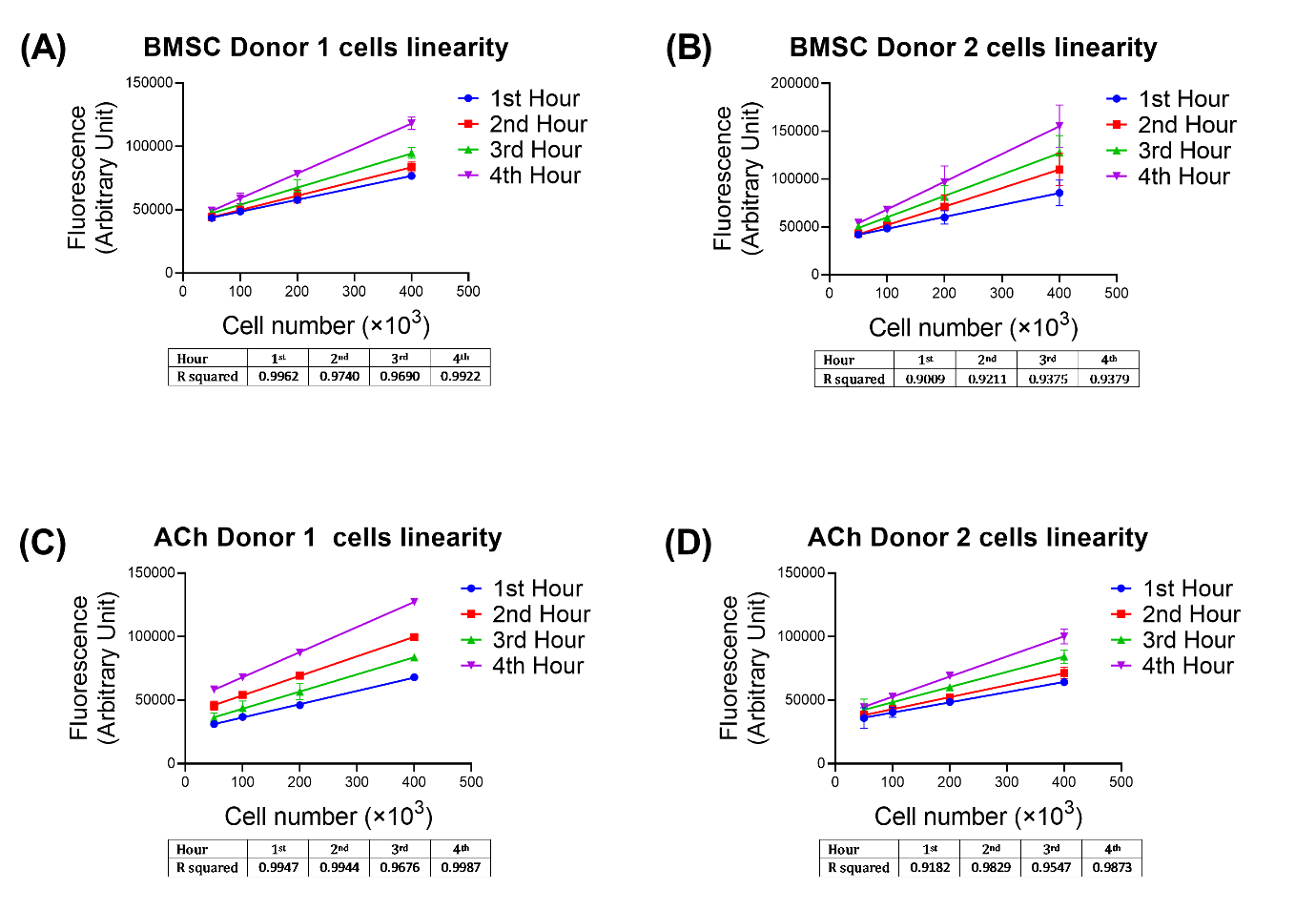


**Supplementary Figure 7**: Linear curves of BMSC and ACh derived cells in AlamarBlue assay. BMSC or ACh cells were seeded into 24 well-plate under chondrogenic media and incubated for 4 hours in 2% O_2_ and 5% CO_2_ at 37°C. After incubation AlamarBlue was diluted to 3% solution into the media and fluorescence reading was measured at 1, 2, 3 and 4 hours. The linear curve was generated by plotting mean percent fluorescence value against cell numbers. **(A)**, **(B)**, **(C)**, and **(D)** represents the linear curves at different time points for BMSC donor 1, BMSC donor 2, ACh donor 1 and ACh donor 2 cells, respectively. Data are presented as mean ± SD.


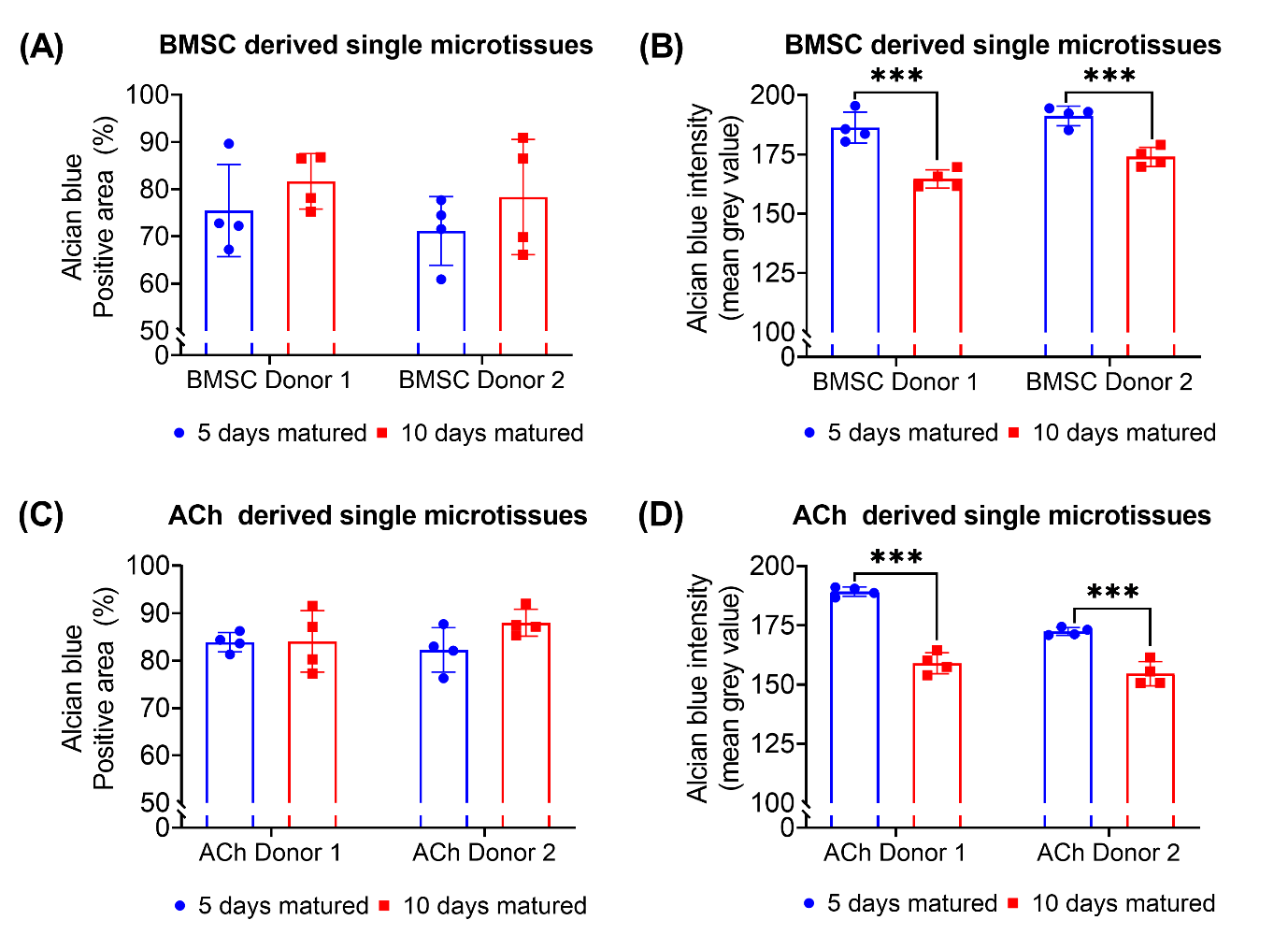


**Supplementary Figure 8**: Quantification of GAG from alcian blue stained BMSC and ACh derived single microtissues. The microtissues were cultured for 5 or 10 days in chondrogenic medium low oxygen (2% O_2_, 5 % CO_2_) at 37 °C. **(A)** and **(C)** represents percentage of positive alcian blue stained area of BMSC and ACh Donors derived microtissues, respectively. **(B)** and **(D)** represents intensity of alcian blue retention of BMSC and ACh Donors derived microtissues, respectively. Data are presented as mean ± SD (n = 4). *** represents *p* < 0.0001 compared to 5 days matured microtissues.


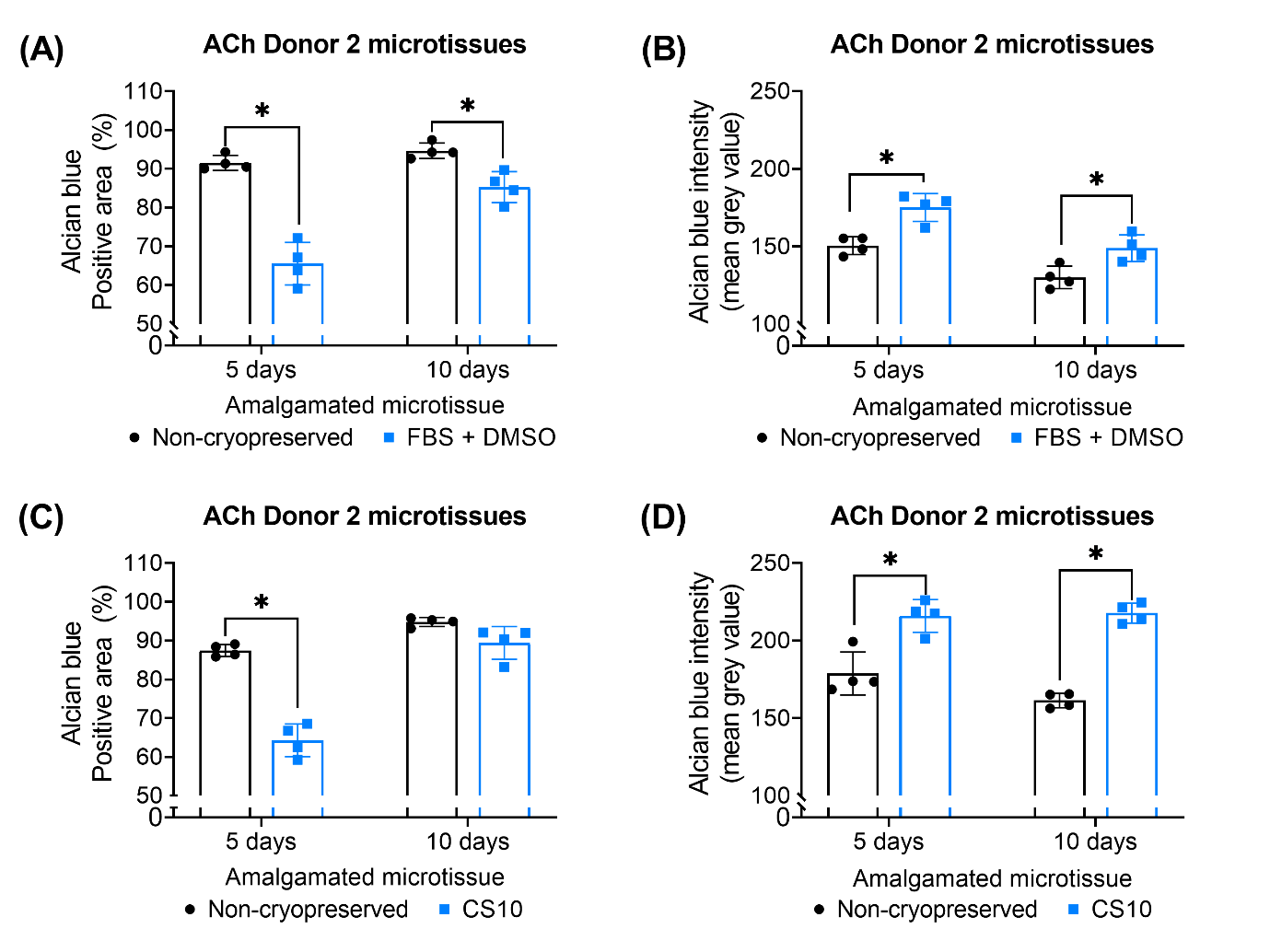


**Supplementary Figure 9**: Quantification of GAG from alcian blue stained ACh Donor 2 derived amalgamated microtissues. ACh Donor 2 microtissues were cultured for 5 or 10 days and cryopreserved in 90% FBS +10 % DMSO or CS10. Cryopreserved BMSC microtissues were thawed and directly washed with chondrogenic medium without any sequential dilution with FBS or Human serum protein. The freshly thawed microtissues were allowed to amalgamate for further 14 days in chondrogenic medium low oxygen (2% O_2_, 5 % CO_2_) or normoxic conditions (20 % O_2_, 5% CO_2_) at 37 °C. **(A)** and **(C)** represents percentage of positive alcian blue stained area of BMSC Donor 1, BMSC Donor 2, derived cryopreserved, and non-cryopreserved amalgamated tissues in different oxygen concentration conditions, respectively. **(B)** and **(D)** represents intensity of alcian blue retention of ACh Donor 1, ACh Donor 2, derived cryopreserved, and non-cryopreserved amalgamated tissues.
